# Protein analysis of boar seminal plasma proteins with protective effect during low-temperature storage of spermatozoa

**DOI:** 10.1080/13102818.2014.926679

**Published:** 2014-10-28

**Authors:** Denica Daskalova, Alexander Kukov, Irina Kirilova, Maria Ivanova-Kicheva

**Affiliations:** ^a^ Department of Reproductive Biotechnology and Cryobiology of Gametes, Institute of Biology and Immunology of Reproduction, Bulgarian Academy of Sciences, Sofia, Bulgaria

**Keywords:** SPPs, chromatography, 2D electrophoresis, low-temperature storage

## Abstract

The present study aimed to investigate the effect of different seminal plasma proteins (SPPs) on boar spermatozoa functional characteristics. We investigated the putative protective effect of SSPs on sperm cells motility and velocity, as well as on the integrity of the plasma membrane (PM) during low-temperature storage at 4 °C. SPPs fractions were obtained and purified by gel permeation chromatography (GPC). Nine fractions of SPPs were obtained and further characterized by 12% sodium dodecyl sulphate polyacrylamide gel electrophoresis (SDS-PAGE). Sperm computer analysis (SCA) after incubation of spermatozoa with separated proteins revealed that fraction 6 consisting of low molecular weight (MW) proteins could preserve spermatozoa motility and velocity better when compared to those with higher MW. Two-dimensional (2D) elecrtophoretic analysis showed that fraction 6 contained proteins with the following MW and isoelectric point (pI): 16 kDa and pI 7.35, 18 kDa and pI 5.20, 19 kDa and pI 7.35, 26 kDa and pI 4.50, 26 kDa and pI 4.30, 29 kDa and pI 5.85.

## Introduction

Seminal plasma (SP) is a composite secretion which contains a lot of proteins originating from the testis, epididymis and accessory glands. The protein composition of mammalian SP varies among species and could affect spermatozoa functions.[1,2] Several seminal plasma proteins (SPPs) have been reported to influence the motility, survival rate and fertility potential of spermatozoa.[2] A putative role of SPPs in prevention of sperm cells from the effect of low temperature has also been suggested.[3] During the process of cooling and cryopreservation, sperm cells undergo changes in motility, plasma membrane (PM) integrity and mitochondrial function. It is now well known that boar spermatozoa are more sensitive to cold shock stress than those of other species such as bull, rabbit or human.[1] It should be noted that the fertilizing ability of boar sperm cells after cold-shock is unsatisfactory.[4] This poses the question of finding out more efficient approaches for spermatozoa protection during storage at low and ultra low temperatures. One promising field of investigations in this direction is the evaluation of boar SPPs as natural protectors which might play a crucial role in spermatozoa viability after cooling. The majority of boar SP proteins belong to the group of spermadhesins,[3,5] which have an important physiological role and participate in the sperm-cell development, including capacitation, interaction of gametes, preparation of the uterus for conception.[6] Some of the spermadhesins express ligand-binding affinity for heparin, phospholipids, carbohydrates and protease inhibitors. The family of these proteins can be divided into heparin-binding (AQN-1, AQN-3, AWN) and non-heparin-binding spermadhesins (PSP-I and PSP-II).[6,7]

It has been shown that ram SPPs with molecular weight (MW) of 14 and 20 kDa could have a protective effect on sperm function after storage at low temperatures.[3,8] In support of this view, the importance of two proteins of boar SP with MW of 26 kDa and isoelectric point (pI) of 6.2 and 55 kDa and pI 4.8 has been recently reported. The authors showed that the high levels of both proteins in boar's ejaculate are corresponding with the high rate of farrowing (over 86%) and numbers of live-born piglets (over 11%).[4,7] Flowers [10] found that inclusion of 10%–12% of SP in the dose for artificial insemination (AI) may have a beneficial effect on the biological parameters of boar semen. The biological effects of SPPs on spermatozoa, functions are still not fully understood.[10,12] This prompted us for further evaluation of the role of boar SPPs on sperm cells motility and viability after cooling.

The aim of the present study was to investigate the effect of SPPs with different molecular weights on the motility and viability of boar spermatozoa after *in vitro* storage at low temperatures (4 °C).

## Materials and methods

### Preparation of samples

Ejaculates were collected from healthy Large White boars. Immediately after collection of semen, sperm motility and concentration, number of live and dead sperm cells, semen pH, survival and morphological analysis were assessed. SP was yielded by centrifugation of boar semen at 2000 r/min, at 4 °C for 5 min. Afterwards, supernatant was collected and again centrifuged at 12,000 r/min for 5 min, then filtered through a 0.22 μm membrane (Millipore) and kept at −80 °C until assay.

### Chromatography separation of seminal plasma

For gel permeation chromatography (GPC), 1 mL of the SP was loaded onto semi-preparative column TSK gel G3000SW, 21.5 mm × 300 mm (TOSOH BIOSCIENCE) at a flow rate of 6 mL/min and nine SPP fractions were obtained. Protein content in the collected samples was determined specrtophotometrically (Ultrospec-200, Pharmacia Biotech,) and visualized by 12% sodium dodecyl sulphate polyacrylamide gel electrophoresis (SDS-PAGE) after silver staining (Garl Roth).

### Evaluation of seminal plasma proteins effect

To evaluate the effect of SPPs on spermatozoa, eight ejaculates from Large White boars were used. After quantification of semen parameters samples were divided into two equal parts, each one was diluted 1:2 with extender CC05 (4.0 g of glucose, 0.27 g of helaton, 0.6 g of NaНCO_3_, 4 g of Na citrate, 1.5 g of (NH_4_)_2_SO_4_, 0.5 g of coffein, 20 mL egg yolk). Semen samples were centrifuged at 2000 r/min for 5 min at room temperature, to exclude the SP. Treated sperm cells were resuspended in CC05 at concentration of 1:4. Resuspended samples were aliquoted into equal volumes and 500 μL of SPPs from fractions 1, 2, 3, 4, 5, 6 and 7 were added to the appropriate sample, to a final volume of 1 mL. Thus, prepared samples were incubated at 4 °C for 24 h. Controls were included as follows: C+ (with whole SP) and C– (without SP); instead of SPPs only extender was added to the C– controls.

### Evaluation of boar sperm motility by sperm computer analyser

Motility of sperm cells was monitored prior to and after incubation at 4 °C for 24 h, the assessment was done by Sperm Class Analyzer (Micropticum, Spain). Measurements were conducted using the Motility&Concentration Software and for each sample the following parameters were recorded: progressive motility (an actual space-gain motility); curvilinear velocity (VCL; shows the rate of sperm for the actual time elapsed from point to point, or distance travelled over a given period of time); straight line velocity (VSL; the straight line distance from beginning to end of a sperm track divided by the time taken) and average path velocity (VAP; the speed of sperm motion for the average distance travelled in space time). Velocity data were expressed in μm/s.

### 6CFDA/PI test for assessment of the plasma membrane integrity

Following incubation with separated SPPs at 4 °C for 24 h, sperm cells were washed twice and resuspended in 1 mL phosphate-buffered saline (PBS) containing etylenediaminetetraacetic acid (EDTA) in a concentration of 1 million cells per millilitre. Ten microliters of carboxyfluorescein diacetate (CFDA; 1 mg/mL in dimethyl sulfoxide (DMSO)) and 5 μL of propidium iodide (PI) (1 mg/mL in PBS) were added to 100 μL of cell suspension. Samples were stained under dark conditions for 30 min at room temperature. The cells were then examined under an Olympus fluorescence microscope with an appropriate filter, and the number of CFDA-positive (live, membrane-intact) spermatozoa and PI-positive (dead, membrane-damaged) spermatozoa per 100 cells were estimated and recorded. At least 200 cells were counted for each sample.

### 2D electorophoresis of fraction 6

Isoelectric focusing of fraction 6 was done to determine with a higher resolution the specific content of proteins. Two-dimensional (2D) electrophoresis was performed using Milltiphor II (Pharmacia) and a pH gradient of 3.0–10.0 (GEHealthcare). Before separation, proteins were purified using 10% trichloroacetic acid in acetone and 0.07% 2-mercaptoethanol (Sigma, SL, USA). The protein samples were centrifuged at 13,000 r/min for 30 min at 4 °С and washed twice with acetone. The samples were dried and after that dissolved in a buffer for isoelectric focusing (8 mol/L urea, 2 mol/L triurea, 0.5% isoelectric point gel (IPG) buffer (3–11) (General Electric Healthcare), 4% 3-((3-cholamidopropyl)dimethylammonio)-1-propanesulfonic acid (CHAPS), 30 mmol/L DL-dithiothreitol (DTT), 20 mmol/L Tris HCl (pH 8.8). Fifty microlitre (40 μg of protein) samples were loaded on strips (Immobiline DryStrips pH 3–11, 13 cm, General Electric Healthcare). Isoelectrofocusing was performed at 500 V, 7 mA, 7 W for 30 min and 2000 V, 7 mA, 7 W for 1.5 h. Corresponding isoelectric points (pI) were established by means of isoelectric focusing markers (GE Healthcare). Determination of the MW of proteins in fraction 6 was performed using 15% SDS-PAGE. MW marker from 6.5 kDa to 200 kDa (Sigma, SL, USA) was loaded into the gels to determine the MW of the representing spots. Proteins were visualized by silver staining (Garl Roth) and the gels were scanned on a Scanner Image Skanneer III, GE Healthcare.

Data are present as means ± standard deviation (SD). Statistical analysis, where necessary, was made with the Student's *t*-test, and the statistical program Motility and Concentration (Microptic, Spain).

## Results and discussion

The semen samples used in the study were odourless, without agglutination, with concentration of 200 × 10^6^ to 400 × 10^6^ sperm cells/mL and with semen pH between 7.3 and 7.8.

### Chromatographic separation of SPPs

To obtain baseline information about the pattern of boar SPPs, SDS-PAGE analysis was performed after the GPC. The analysis revealed nine major SPP fractions in the SP. The protein concentration measured spectrophotometrically varied from 0.010 to 2.068 mg/mL. The protein content appeared with different molecular weight, ranging from 10 to 200 kDa. Fraction 1, 2 and 3 contained SPPs with high MW from 55 to 200 kDa, fraction 4 and 5 contained proteins with MW from 20 to 97 kDa, and low MW proteins (up to 30 kDa) were found in samples 6 and 7.

### Effect of low temperatures on sperm velocity parameters

Notably, sperm motility was influenced in a different manner in the samples after incubation of boar spermatozoa at 4 °C for 24 h with separated SPPs. Cooling resulted in a significant reduction of total motility as assessed by the sperm computer analyser. The obtained data are shown in Table 1. The percentage of static cells was significantly higher in samples that contained SPPs from fractions 2, 3, 4 and C– than in the samples C+ and 6 (*P* < 0.05 and *P* < 0.001). The percentage of progressive motile spermatozoa in samples 6 and C+ was 51.00 ± 5.4 and 43.70 ± 9.49, respectively, while in samples C–, 1 and 3 it was 14.63 ± 2.06, 31.60 ± 7.18 and 30.32 ± 5.53, respectively, with a statistically significant difference between samples 6 and 3 (*P* < 0.05).

**Table 1.** Sperm motility after low-temperature storage.

Samples	Progressive motile (%)	Non-progressive motile (%)	Static (%)
C+	43.70 ± 9.49^а^	40.75 ± 5.70^a^	15.55 ± 4.05^c^
C−	14.63 ± 2.06^c^	26.31 ± 4.29^c^	59.08 ± 2.32^а^
Fraction 1	31.60 ± 7.18^b^	42.63 ± 4.01^c^	25.77 ± 4.09^b^
Fraction 2	28.76 ± 8.51^b^	39.01 ± 3.06^c^	32.23 ± 5.04
Fraction 3	30.32 ± 5.53^b^	39.20 ± 8.28	30.48 ± 10.49
Fraction 4	33.28 ± 5.62	32.1 ± 5.77	34.65 ± 11.09
Fraction 5	39.73 ± 11.72	40.54 ± 6.17	19.73 ± 4.10^c^
Fraction 6	51.00 ± 5.41^а^	32.05 ± 2.11^a^	17.95 ± 3.64^c^
Fraction 7	37.25 ± 6.92	41.18 ± 6.70	21.57 ± 1.34^b^

Note: Significant differences shown between ^a^ and ^b^ at *P* < 0.05; between ^a^ and ^c^ at *P* < 0.001.

Data of velocity parameters (VCL, VSL and VAP) after incubation at 4 °C for 24 h are shown in Table 2. Analysis revealed that in all samples the mean values decreased significantly during low-temperature storage. The higher values of VCL were in samples C+ and in samples incubated with fraction 6 in contrast to parameters found in samples C–, 1, 2, 3 and 7. The differences between samples C+, 6 and C– were statistically significant (*P* < 0.001). VSL parameters were lower in sample C– compared to fraction 6. VAP was lower in the samples without SP and in the samples with spermatozoa that were incubated with high MW SPPs.

**Table 2.** Sperm velocity parameters (VCL, VSL and VAP).

Samples	VCL (μm/s)	VSL (μm/s)	VAP (μm/s)
C+	46.56 ± 5.39^а^	19.10 ± 8.24	31.65 ± 9.48^а^
C−	14.63 ± 2.84^c^	8.63 ± 1.29^b^	13.63 ± 1.69^b^
Fraction 1	39.96 ± 12.64	12.73 ± 3.35	18.60 ± 3.78
Fraction 2	34.90 ± 2.25	18.61 ± 4.57	25.69 ± 4.97
Fraction 3	36.87 ± 11.18	12.30 ± 2.11	21.85 ± 5.42
Fraction 4	39.96 ± 6.90	15.25 ± 2.47	26.08 ± 4.77
Fraction 5	33.93 ± 3.55	11.70 ± 3.01^б^	19.40 ± 3.11
Fraction 6	47.25 ± 4.64^а^	26.75 ± 2.16^а^	23.85 ± 3.10
Fraction 7	27.35 ± 6.33	13.00 ± 1.90^b^	21.88 ± 2.40

Note: Significant differences shown between ^a^ and ^b^ at *P* < 0.05; between ^a^ and ^c^ at *P* < 0.001.

### Assay of plasma membrane integrity

The viability of sperm cells after *in vitro* exposure to SPPs was analysed by CFDA/PI double staining. Assessment of boar sperm PM integrity by fluorescent markers after incubation with different fractions of separated SPPs at 4 °C for 24 h revealed specific influence exerted by the proteins in fractions 1, 2, 3 and 6. Higher number of PM damaged sperm cells (PI+ stained) was observed in C– (70.95 ± 5.49) and in samples incubated with high-MW proteins 1, 2 and 3 (43.30 ± 7.69; 65.56 ± 4.76 and 44.57 ± 8.88, respectively). The highest number of CFDA-positive, live spermatozoa was found in samples incubated either with whole SP (C+) or with fraction 6 (63.74 ± 7.14 and 73.65 ± 6.68, respectively), whereas in the samples incubated with fractions containing high-MW proteins the percent of CFDA-positive sperm cells was low overall. The lowest value of CFDA+ spermatozoa was found in the control C– (29.05 ± 6.18) incubated in the absence of SP.

**Figure 1.**
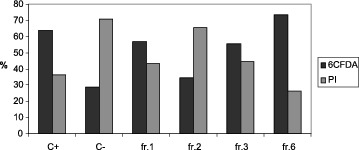
Representative CFDA/PI double staining of boar spermatozoa incubated at 4 °C for 24 h, with different fractions of seminal plasma proteins.

The percentage of viable sperm cells was significantly higher in sample 6 (73.65 ± 7.76) in comparison with samples C–, 2 and 3 (29.05 ± 6.18, 56.70 ± 3.44, 34.44 ± 3.67 and 55.43 ± 5.55 respectively) as shown in Figure 1.

The obtained results indicated that the storage of spermatozoa at low temperature resulted in a significant reduction of the integrity of the PM and in a percentage decrement of progressive motile spermatozoa. Our results coincide with the findings of other authors that address the relevance of specific SPPs for the function and viability of sperm cells.[2,3,8] We have demonstrated a protective effect of certain class of separated SPPs on sperm functions which may be useful in the development of new extenders for *in vitro* storage and cryopreservation of boar spermatozoa.

As shown in Table 1, Table 2 and Figure 1, we observed an increased number of PI-positive spermatozoa in the samples which contained high MW SPPs. This finding supports previous reports showing that high MW factors might induce capacitation and destabilization of sperm cells PM during prolonged *in vitro* storage and lead to spermatozoa injury.[5,10] We found that after cooling at 4 °C the percentage of viable spermatozoa (CFDA+) was highest in samples incubated with the fraction 6 obtained by GPC and further characterized by 2D electrophoresis.

### 2D electorophoresis of fraction 6

Classically, 2D electrophoresis performed as a combination of isoelectric focusing and SDS-PAGE has been the only method used to analyse the proteome with high resolution.[12] In our study, 2D electrophoresis was utilized to explore the protein contained in SP faction obtained after preparative GPC. To obtain more information on the protein content of fraction 6, 2D electrophoretic analysis of the fraction was carried out.

**Figure 2.**
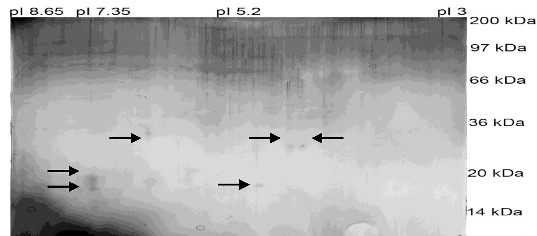
Silver-stained 2D electrophoresis of fraction 6 isolated from boar seminal plasma by gel permeation chromatography.

Six protein spots were visualized on the gel which corresponded to proteins with the following MW and isoelectric points: 18 kDa and pI 5.20; 26 kDa and pI 4.50; 26 kDa and pI 4.30; 29kDa and pI 5.85, 19 kDa and pI 7.35, 16 kDa and pI 7.35 (Figure 2). Two proteins appeared with different MW (16 and 19 kDa), but with the same isoelectric points (pI 7.35), and two others had the same MW of 26 kDa but showed different isoelectric points (pI 4.50 and pI 4.30). We were able to identify six protein spots on the gel. We suggested that the protein identified as 26 kDa and pI 4.50, belongs to the family of retinolbinding proteins.[9] Other proteins, with 16 kDa and pI 7.35, 18 kDa and pI 5.20 and 19 kDa and pI 7.35, may belong to the large group of boar SSPs called spermadhesins.[10]

Recently, it has been demonstrated that proteins from ram SP with low MW may protect the sperm PM and revert the damage of spermatozoa caused by cold shock.[3] It has been already shown that the addition of definite SPPs may protect sperm cells.[1,11,12]

We found that boar spermatozoa incubated in extenders which contained low MW SPPs from 16 kDa to 29 kDa retain better progressive motility, VCL, VSL and VAP parameters, as well as PM integrity. These proteins and the two others with 26 kDa and pI 4.30 and 29 kDa and pI 5.85 may be candidates for protective agents during low-temperature storage of boar semen. We suppose that the effect of these proteins might be exerted by adhesion in specific regions оf the PM. By means of adhesion the proteins may stabilize the membrane and most probably might prevent damages caused by low-temperature storage. Spermatozoa with stabilized PM showed better viability and motility of boar spermatozoa and most probably have retained fertilizing ability. Our results indicated that isolated SPPs contained in sample 6 retained the greatest number of spermatozoa with well-preserved PM in comparison with other samples after storage at 4 °C for 24 h. Consequently, the presence of SPPs with MW lower than 30 kDa (fraction 6) may protect boar spermatozoa through preservation of PM integrity and sperm velocity parameters. This fraction may prove to be a promising candidate for a protective agent in media designed for storage of boar semen.

## Conclusions

In conclusion, our results indicated that the incubation of boar spermatozoa with separated low MW SPPs (16–29 kDa) better preserved the motility, velocity parameters (VCL, VSL, VAL) and the integrity of PM of the germ cells. The addition of these SPPs as biological components to protective media may find application in the formulation of better extenders for preserving boar semen during low-temperature storage at 4 °C and manipulations associated with cold shock.
